# Urinary continence outcomes after robot‐assisted laparoscopic radical prostatectomy: Significance of anterior reconstruction

**DOI:** 10.1111/iju.15654

**Published:** 2024-12-23

**Authors:** Keisuke Funajima, Sei Naito, Atsushi Fukai, Takafumi Narisawa, Hiroki Fukuhara, Shinta Suenaga, Yuki Takai, Satoshi Takai, Mayu Yagi, Hidenori Kanno, Atsushi Yamagishi, Hayato Nishida, Norihiko Tsuchiya

**Affiliations:** ^1^ Department of Urology Yamagata University Faculty of Medicine Yamagata Yamagata Prefecture Japan

**Keywords:** continence, postoperative complications, prostate cancer, robot‐assisted laparoscopic radical prostatectomy

## Abstract

**Objective:**

Urinary continence after radical prostatectomy is a crucial aspect of patient quality of life. The aim of this study was to identify the factors influencing urinary continence after robot‐assisted laparoscopic radical prostatectomy, focusing on the role of anterior reconstruction.

**Methods:**

We collected clinical data from 375 patients at a single institution. Logistic regression analyses for urinary continence rate at 1, 3, 6, and 12 months postoperatively were performed on the entire patient population to determine the influencing factors. Anterior and posterior reconstruction was performed until August 2017, transitioning to posterior reconstruction only. The impact of anterior reconstruction on postoperative urinary continence was evaluated using logistic regression model adjusted by inverse‐probability treatment weighting in nerve‐sparing and non‐nerve‐sparing subgroups, respectively.

**Results:**

For the entire cohort, the urinary continence rates at 1, 3, 6, and 12 months were 34.7%, 57.6%, 73.1%, and 83.5%, respectively. Anterior reconstruction significantly influenced early urinary continence recovery, and membrane urethral length correlated with continence rates at all postoperative time points. After adjustment using the IPTW method, the chronological trend of urinary continence recovery rate in relation to anterior reconstruction was similar between patients with and without nerve sparing.

**Conclusions:**

Anterior reconstruction contributes to early recovery from urinary incontinence after robot‐assisted laparoscopic prostatectomy. However, the impact for continence rate 12 months after surgery is limited.

**Abbreviations & Acronyms**
95% CI: 95% confidence interval
BMI: body mass index
IPTW: inverse probability of treatment weighting
IQR: interquartile range
LND: lymph node dissection
LRP: laparoscopic radical prostatectomy
MUL: membrane urethral length
OR: odds ratio
ORP: open radical prostatectomy
PSA: prostate‐specific antigen
RARP: robot‐assisted laparoscopic radical prostatectomy
RP: radical prostatectomy

## INTRODUCTION

Radical prostatectomy (RP) is one of the treatment options applicable to localized prostate cancer patients with an expected prognosis of more than 10 years.^1^, ^2^, ^3^, ^4^ Open radical prostatectomy (ORP) has evolved into laparoscopic radical prostatectomy (LRP) and robot‐assisted laparoscopic radical prostatectomy (RARP). RARP is recommended as a safer and less invasive surgical procedure with equivalent oncologic efficacy compared to ORP and LRP.^5^, ^6^, ^7^

Urinary incontinence after RP significantly affects patients' quality of life,^8^ and RARP shows better recovery of urinary continence compared to ORP and LRP. Previous reports have shown that urinary incontinence improves in 60%–96% of cases after ORP, whereas in approximately 90% of cases after RARP.^6^, ^9^, ^10^, ^11^

Postoperative urinary incontinence is influenced by various complex factors. Preoperative factors reported include age, body mass index (BMI), comorbidities, erectile function, and the length of membrane urethra length (MUL).^9^, ^12^ Surgical techniques involving nerve‐sparing, sphincter preservation, bladder neck preservation, and pelvic floor reconstruction also play a role in postoperative continence.^9^, ^11^, ^13^, ^14^, ^15^, ^16^ However, previous reports on pelvic floor reconstruction have not evaluated the efficacy of anterior and posterior reconstruction compared to posterior reconstruction alone.^11^, ^17^, ^18^, ^19^ In this study, we retrospectively evaluated the factors influencing urinary continence recovery after RARP at our institution, with a particular focus on the impact of anterior reconstruction in patients with posterior reconstruction.

## PATIENTS AND METHODS

### Patients

Clinical data were collected from our institution's electronic medical records. We used data from patients who underwent RARP from July 2012 to July 2020. A total of 15 surgeons performed RARP. Indications for RARP at our hospital were cases with cT3 or less and no metastasis. We performed nerve‐sparing on the side where there was no prostate cancer in the peripheral zone on prostate biopsy, but the decision to actually perform the procedure was based on the patient's preference. Lymph node dissection (LND) was performed for patients whose Briganti score predicted a >15% probability of lymph node metastasis. In most cases, RARP with LND was performed by surgeons with an experience of more than 50 cases.

### Surgical technique

Patients were placed in the lithotomy position with the head lowered at 25°. The da Vinci S and Si^®^ Surgical System was used, configured with six trocars and a four‐arm approach. The Retzius cavity was expanded using a transabdominal approach. Puboprostatic ligaments were cut and the dorsal venous complex (DVC) was sutured with 3‐0V‐Lock^®^ after cutting the DVC. Preservation of the bladder neck was not intended, but the urethra was maximally preserved in oncologically safe cases. After removal of the prostate, posterior reconstruction was performed using the Rocco technique with suturing rhabdo‐sphincter, median raphe, and denovillier's fascia in all cases.^20^ The vesicourethral anastomosis was sutured continuously with two 3‐0V‐Lock^®^. Anterior reconstruction was then performed with continuous 3‐0V‐Lock^®^ sutures from the anterior pubovesical collar and DVC or puboprostatic ligament. Although posterior reconstruction was performed in all patients during the study period, anterior reconstruction was performed in all patients until August 2017, but was omitted in most cases after September 2017. The indwelling urethral balloon catheter was removed on postoperative day 7.

### Statistical analysis

Based on previous reports, age, BMI, preoperative prostate volume, nerve preservation, LND, MUL, and anterior reconstruction were candidate factors contributing to postoperative urinary incontinence. Continence recovery was evaluated at 1, 3, 6, and 12 months postoperatively. Recovery of continence was defined as not using a pad or using only a safety pad. Logistic regression was performed for the total patient population. The effects of anterior reconstruction on postoperative urinary continence were evaluated using a *χ* ^2^ test, adjusting for differences in age, BMI, LND, MUL, and preoperative prostate volume using the inverse‐probability treatment‐weighted (IPTW) method in the nerve‐sparing and non‐nerve‐sparing subgroups, respectively. Differences in patient background were evaluated using standard mean difference (SMD). No difference was considered when the SMD was <0.25. All statistical analyses were performed using statistical software R version 4.3.1 and its packages; survey, RISCA, survival, and EZR version 1.63 (Saitama Medical Centre, Jichi Medical University; http://www.jichi.ac.jp/saitama‐sct/SaitamaHP.files/statmedEN.html;Kanda,2012).^21^

## RESULTS

### All cohort

**FIGURE 1:**
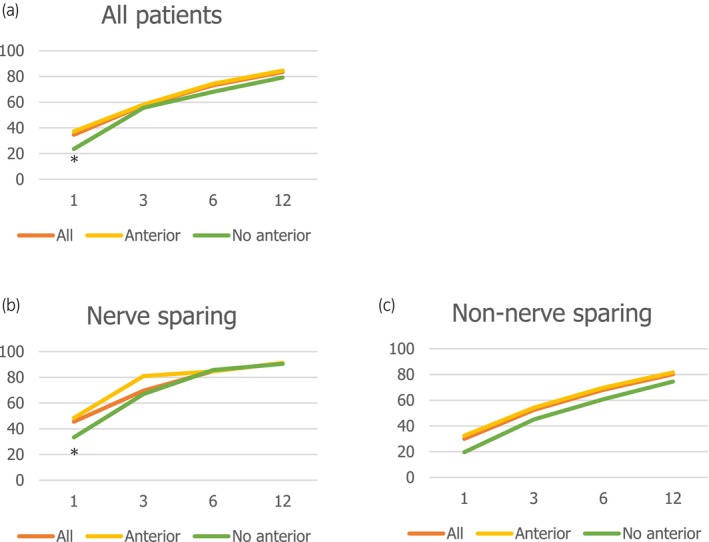
(a) Urinary continence rate in all cohort. (b) Urinary continence rate in nerve‐sparing cases. (c) Urinary continence rat in non‐nerve‐sparing cases. *p < 0.05 for comparison with and without anterior reconstruction.

Baseline characteristics are shown in Table 1. Because urinary continence data were missing in 18 patients and MUL data were missing in 44 patients, we evaluated 375 of 437 patients who underwent RARP at our institution between 2012 and 2020. Among them, 303 (80.8%) patients received anterior reconstruction and 112 (30.0%) patients received nerve‐sparing technique. There was no apparent difference in operative time among these surgical techniques. The urinary continence recovery rate at 1, 3, 6, and 12 months after RARP was 34.7%, 57.6%, 73.1%, and 83.5%, respectively (Figure 1a). In logistic regression analysis, the factors contributing to continence recovery at 1 month after RARP were anterior reconstruction (OR = 2.07, 95% CI 1.11–2.63, *p* = 0.023), LND, nerve‐sparing (OR = 1.72, 95% CI 1.04–2.84, *p* = 0.035), and long MUL (OR = 1.08, 95% CI 1.03–1.13, *p* < 0.001). While MUL and LND had high ORs over time after RARP, anterior reconstruction was not statistically independent after 3 months. Age and BMI were associated with continence rate at 3 months (OR [95% CI] = 0.95 [0.91–0.99] in age and OR [95% CI] = 0.92 [0.85–0.99] in BMI). Prostate volume did not show statistical difference at any time points (Table 2).

**TABLE 1:** Patient characteristics.

	All (*N* = 375)	Nerve‐sparing (*N* = 112)	Non‐nerve‐sparing (*N* = 263)	Anterior reconstruction (*N* = 303)	Non‐anterior reconstruction (*N* = 72)
Age, years, median (IQR)	67 (63–70)	65 (60–68)	68 (64–71)	67 (62–71)	66 (63–70)
BMI, median (IQR)	23.7 (22.0–25.5)	23.4 (21.6–25.3)	23.7 (22.1–25.4)	23.7 (22.0–25.4)	23.3 (22.1–25.2)
PSA, ng/mL, median (IQR)	7.3 (5.5–10.6)	6.7 (5.4–9.7)	7.7 (5.5–10.9)	7.17 (5.37–10.2)	9.11 (6.34–11.9)
Prostate volume, g, median (IQR)	32.0 (23.4–42.0)	35.0 (23.0–43.6)	31.0 (23.0–39.5)	32 (24–41)	30 (23–44)
MUL, mm, median (IQR)	12.2 (9.0–15.9)	12.9 (9.6–17.9)	11.7 (8.7–15.6)	11.8 (8.9–15.9)	13.1 (10.1–16.3)
cT stage, *N* (%)					
1c	63 (16.8)	29 (25.9)	34 (12.9)	54 (17.8)	9 (12.5)
2a	173 (46.1)	58 (51.8)	115 (43.7)	137 (45.2)	36 (50.0)
2b	40 (10.7)	10 (8.9)	30 (11.4)	31 (10.2)	9 (12.5)
2c	75 (20.0)	12 (10.7)	63 (24.0)	64 (21.1)	11 (15.2)
3a	21 (5.6)	1 (0.9)	20 (7.6)	17 (56.7)	4 (5.6)
3b	2 (0.5)	1 (0.9)	1 (0.4)	0	2 (2.8)
Unidentified	1 (0.3)	1 (0.9)	0	0	1 (1.4)
pT stage, *N* (%)					
2	273 (72.8)	95 (84.8)	178 (68.2)	221 (73.2)	52 (73.2)
3	97 (25.9)	16 (14.3)	81 (31.0)	79 (26.2)	18 (25.4)
4	1 (0.3)	1 (0.9)	0	0	1 (1.4)
Unidentified	4 (1.1)	0	2 (0.8)	2 (0.7)	0
Gleason score, *N* (%)					
≤6	85 (22.7)	35 (31.3)	50 (19.0)	71 (23.4)	14 (19.4)
7	182 (48.5)	61 (54.5)	121 (46.0)	150 (49.5)	32 (44.4)
≥8, *N*	108 (28.8)	16 (14.3)	92 (35.0)	82 (27.1)	24 (33.3)
Anterior reconstruction, *N* (%)					
Done	303 (80.8)	91 (81.2)	212 (80.6)		
None	72 (19.2)	21 (18.8)	51 (19.4)		
LND, *N* (%)					
Done	179 (47.7)	46 (41.1)	133 (50.6)	156 (51.5)	23 (31.9)
None	196 (52.3)	66 (58.9)	130 (49.4)	147 (48.5)	49 (68.1)
Operative time, min, median (IQR)					
All	222 (187–253)	223 (189–253)	221 (187–254)		
Anterior reconstruction	222 (188–251)	224 (189–255)	220 (188–247)		
Non‐anterior reconstruction	227 (187–273)	223 (191–245)	238 (181–285)		

Abbreviations: BMI, body mass index; IQR, interquartile range; LND, lymph node dissection; MUL, membrane urethral length; PSA, prostate‐specific antigen.

**TABLE 2:** Logistic regression analysis of urinary continence rate at 1, 3, 6, and 12 months after RARP in all cohort.

	1 month			3 months			6 months			12 months		
	OR	95% CI	*p*	OR	95% CI	*p*	OR	95% CI	*p*	OR	95% CI	*p*
Age, year	0.99	0.95–1.03	0.464	0.96	0.92–1.00	0.067	0.97	0.93–1.02	0.226	0.97	0.92–1.03	0.299
BMI, kg/m^2^	0.92	0.85–1.00	0.055	0.91	0.84–0.98	0.017	0.96	0.88–1.05	0.386	0.91	0.82–1.01	0.068
Prostate volume, mL	1.00	0.99–1.02	0.885	1.00	0.98–1.01	0.760	0.99	0.98–1.01	0.310	0.99	0.97–1.01	0.160
MUL, mm	1.08	1.03–1.13	<0.001	1.10	1.05–1.16	<0.001	1.12	1.06–1.19	<0.001	1.13	1.06–1.22	<0.001
Anterior reconstruction	2.07	1.11–3.86	0.023	1.10	0.63–1.95	0.725	1.32	0.72–2.44	0.373	1.39	0.69–2.83	0.360
LND	1.65	1.03–2.63	0.036	2.21	1.39–3.50	<0.001	2.32	1.39–3.90	0.001	2.62	1.39–4.91	0.003
Nerve sparing	1.72	1.04–2.84	0.035	1.72	1.02–2.89	0.040	2.22	1.19–4.17	0.013	2.05	0.94–4.47	0.072

Abbreviations: BMI, body mass index; LND, lymph node dissection; MUL, membrane urethral length; OR, odds ratio; RARP, robot‐assisted laparoscopic radical prostatectomy.

### Nerve‐sparing group

As a subgroup analysis, we investigated whether anterior reconstruction contributed to the recovery of urinary continence in both nerve‐sparing and non‐nerve‐sparing groups. In the nerve‐sparing group, anterior reconstruction tended to improve urinary continence at 1 month, but no difference was observed after 3 months (Figure 1b). On IPTW analysis adjusted for age, BMI, MUL, LND, and PV (Table S1), the OR was high in anterior reconstruction group only at 1 month (Table 3).

**TABLE 3:** IPTW adjusted OR of urinary continence rate at 1, 3, 6, 12 months after RARP in nerve‐sparing cohort.

	OR	95% CI	*p*
1 month	3.25	1.07–9.86	0.038
3 months	0.38	0.11–1.32	0.128
6 months	0.83	0.22–3.22	0.791
12 months	1.26	0.28–5.68	0.759

Abbreviations: 95% CI, 95% confidence interval; IPTW, inverse probability of treatment weighting; OR, odds ratio; RARP, robot‐assisted laparoscopic radical prostatectomy.

### Non‐nerve‐sparing group

In the non‐nerve‐sparing group, anterior reconstruction appeared to contribute to recovery from urinary incontinence at any time points. On IPTW‐adjusted analysis (Table S2), although anterior reconstruction was not associated with recovery from urinary incontinence at any time points, the OR was relatively higher at 1 month (Table 4).

**TABLE 4:** IPTW adjusted OR of urinary continence rate at 1, 3, 6, 12 months after RARP in non‐nerve‐sparing cohort.

	OR	95% CI	*p*
1 month	1.89	0.91–3.94	0.088
3 months	1.46	0.79–2.68	0.226
6 months	1.44	0.77–2.70	0.259
12 months	1.31	0.63–2.73	0.472

Abbreviations: 95% CI, 95% confidence interval; IPTW, inverse probability of treatment weighting; OR, odds ratio; RARP, robot‐assisted laparoscopic radical prostatectomy.

## DISCUSSION

The effects of pelvic floor reconstruction on urinary continence after RARP remain controversial issue. In addition, there are several methods of pelvic floor reconstruction. Rocco et al. reported that a posterior reconstruction technique with suturing of the rhabdosphincter, median fibrous raphe, and posterior bladder wall reduced continence rate up to 3 months.^20^ However, two randomized controlled trials (RCTs) showed no improvement with posterior reconstruction.^22^, ^23^ The first RCT on periprostatic tissue reconstruction, reported by Menon et al., showed no improvement in early continence rate.^24^ Sammon et al. also reported that a periprostatic tissue reconstruction did not improve urinary continence at both early and 2‐year follow‐up.^25^ However, the later RCTs and meta‐analysis demonstrated the continence benefits of anterior and posterior pelvic floor reconstruction.^17^, ^18^, ^26^ More recently, Student et al. demonstrated that advanced reconstruction of vesicourethral support improves early and 1‐year continence outcomes in an RCT.^27^ In our institution, anterior and posterior pelvic floor reconstruction was performed in early period, and then, anterior reconstruction was omitted. Overall, in this study, anterior pelvic floor reconstruction improved continence rate only at 1 month after RARP (Figure 1a; Table 2).

Previous studies have only compared anterior and posterior pelvic floor reconstruction with no reconstruction, not with posterior reconstruction. In addition, most patients in these studies underwent nerve‐sparing procedures, and no study has demonstrated the benefits of anterior and posterior reconstruction separately in nerve‐sparing and non‐nerve‐sparing patients. In this study, the contribution of anterior reconstruction was evaluated separately for nerve‐sparing and non‐nerve‐sparing groups, and the results were similar in both groups (Tables 3 and 4). Based on these findings, we now perform anterior reconstruction in all cases.

There are two methods of anterior reconstruction. Sugimura et al. reported the two‐layer anastomosis, where the puboprostatic ligament and the anterior pubovesical collar were sutured.^28^ In this study, Sugimura's method was used. Patel et al. reported an anterior suspension, where the suture is placed between DVC and pubic bone.^29^ Although the mechanisms of improvement from urinary continence after RARP with anterior reconstruction remain unclear, Chen et al. demonstrated that two‐layer anastomosis resulted in a wider bladder neck angle, higher bladder neck level, and more oblate bladder, which means preservation of maximum urethral length.^19^ In addition, according to Patel et al., anterior support provides anatomical support for the urethra, which allows the urethra or the rhabdosphincter to be stabilized in its anatomical position.^29^ Although the mechanism by which nerve‐sparing contributes to continence is also still unclear, some authors have described that aberrant intrapelvic somatic nerves in the neurovascular bundle innervate a portion of the rhabdosphincter.^14^

This study has several limitations. First, this study was a retrospective study, which included selection bias in patients who did not undergo anterior reconstruction because of retrospective study. However, our surgical proficiency in the period of anterior reconstruction was not performed in all patients is considered better than in the period of all patients received anterior reconstruction. Second, this study was conducted at a single institution; therefore, it may not be possible to generalize. Third, RARP was performed by 15 surgeons, including novices, and as a result, there was a large difference in the maturity of the surgeon. The operation time was slightly shorter in the cases with anterior reconstruction, which may be related to the maturity of the surgeons. In addition, the continence recovery rate was better in the cases with LND, which is thought to be due to the maturity of the surgeons. However, anterior reconstruction was an independent better factor for urinary continence recovery at 1 month, even when LND was taken into account. Fourth, international consultation on incontinence questionnaire might be better than pad count for estimating recovery of urinary continence. However, we did not test this.

In conclusion, anterior pelvic floor reconstruction contributes early urinary continence recovery after RARP compared to only posterior reconstruction technique.

## AUTHOR CONTRIBUTIONS

**Keisuke Funajima:** Conceptualization; methodology; writing – original draft; investigation; formal analysis. **Sei Naito:** Conceptualization; methodology; investigation; writing – review and editing; formal analysis. **Atsushi Fukai:** Data curation. **Takafumi Narisawa:** Data curation. **Hiroki Fukuhara:** Data curation. **Shinta Suenaga:** Data curation. **Yuki Takai:** Data curation. **Satoshi Takai:** Data curation. **Mayu Yagi:** Data curation. **Hidenori Kanno:** Data curation. **Atsushi Yamagishi:** Data curation. **Hayato Nishida:** Data curation. **Norihiko Tsuchiya:** Supervision; conceptualization.

## APPROVAL OF THE RESEARCH PROTOCOL BY AN INSTITUTIONAL REVIEWER BOARD

Yamagata University Faculty of Medicine Review Board approved the present study (Approval number: 2024‐65).

## INFORMED CONSENT

Written informed consent for participation in the study was waved by using the opt‐out method.

## REGISTRY AND THE REGISTRATION NO. OF THE STUDY/TRIAL

N/A.

## ANIMAL STUDIES

N/A.

## CONFLICT OF INTEREST

Norihiko Tsuchiya and Sei Naito are editorial members of the International Journal of Urology. However, we are not involved in the process of deciding whether or not to accept this study. The other authors declare no conflict of interest.

## Supporting information


Table S1.

Table S2.
